# Kinetics of *L*-ascorbic acid degradation and non-enzymatic browning development in hot-compressed water

**DOI:** 10.3389/fnut.2022.1022254

**Published:** 2023-01-12

**Authors:** Liang Feng, Yan Yang, Ya-ting Xie, Shuang-shuang Liu, Xuan Peng, Sheng Hu, Ai-nong Yu

**Affiliations:** ^1^School of Chemistry and Environmental Engineering, Hubei Minzu University, Enshi City, Hubei, China; ^2^Key Laboratory of Biologic Resources Protection and Utilization of Hubei Province, Enshi City, Hubei, China; ^3^Hubei Provincial Key Laboratory of Occurrence and Intervention of Rheumatic Diseases, Hubei Minzu University, Enshi City, Hubei, China

**Keywords:** hot-compressed water, kinetics, mechanism, *L*-ascorbic acid, degradation

## Abstract

The effect of reaction conditions, which comprised the reaction temperature (150–190°C), processing time (0.50, 0.75, 1.00, 1.25, 1.50, 2.00, and 2.50 h), pH (5.0, 7.0, and 9.5), and concentration (0.03–0.07 mol/L) of *L*-ascorbic acid (ASA), on the degradation of ASA was investigated in hot-compressed water (HCW). The degradation kinetics of ASA and generation kinetics of browning products (BPs) were studied. The results showed that ASA degradation conformed to the pseudo-first-order kinetics, and the formation of BPs was closely related to the concentration of H_3_O^+^ in HCW. The acidic condition (pH = 5.0) and lower concentration of ASA (0.03 mol/L) were more favorable for ASA degradation. In HCW, the average apparent activation energy (*Ea*) of ASA was 15.77, 31.70, and 47.53 kJ/mol at pH 5.0, 7.0, and 9.5, respectively. The possible degradation mechanisms of ASA and the generation of BPs in HCW were proposed based on the experimental results.

## 1. Introduction

*L*-ascorbic acid (ASA) is vitamin C, which is an essential nutrient for humans. There is abundant ASA in fruits and vegetables, and it also exists as a food additive in various processed foods (1–3). The degradation of ASA commonly occurs during the thermal processing of food, which not only leads to the loss of nutrients but also initiates the non-enzymatic browning reactions, resulting in a color change in the food (4–5). There are some related studies reported on the kinetics of the degradation of ASA (6–15). Most of the studies were usually conducted under conventional conditions in which temperature was below 150°C and pressure was at atmospheric pressure (16–18). The temperature and pressure of extracting bioactive compounds from vegetable sources are generally higher than those of experimental conditions, usually above 150°C and much higher pressure (19, 20).

Hot-compressed water (HCW) is also known as subcritical water (4, 21, 22). It generally refers to the liquid state of heated water by controlling the pressure of the system. The temperature of HCW is lower than the critical point (*T*_*c*_ = 373°C, *p*_*c*_ = 22.1 MPa), while it is higher than the boiling point (*T* = 100°C, *p* = 0.1 MPa) (23, 24). Approaching critical conditions, the polarity of HCW drops sharply, and it has some organic solvent properties (25, 26), while retaining the advantages of being nontoxic, environmentally friendly, and economical. Therefore, HCW has been used in many fields, such as extraction of natural products, detection of pesticide residues, degradation of pollutants, and decomposition of woody biomass or lignocellulosic materials. The extraction of antioxidants from the potato skin was carried out in subcritical water (220°C, 2.317 Mpa) (27). The lignocellulosics converted into energy and chemicals were studied by using the semi-flow hot-compressed water treatment (230–270°C, 10 Mpa) (28).

During the extracting processing, such as the extracting bioactive compounds from the vegetable sources, HCW plays an important role. The study of the degradation of ASA and the browning reaction has drawn more attention. However, there is limited research on the degradation kinetics of ASA in HCW. The objective of this study was to elucidate the degradation process kinetics of ASA and to reveal the underlying mechanisms of the browning reaction in HCW. The browning kinetics of ASA and the possible formation mechanisms of BPs were also discussed based on the experimental results and the theoretical explanations. The study on the degradation behavior of ASA in HCW would help to obtain more knowledge about HCW technology.

## 2. Materials and methods

### 2.1. Materials

*L*-ascorbic acid (≥99.7 %) and HPO_3_ were purchased from Sigma-Aldrich Chemical Co. (St. Louis, MO, USA). Na_2_HPO_4_ (≥98.5 %), NaH_2_PO_4_ (≥98.5 %), and NaOH (≥98.5 %) were purchased from Sinopharm Chemical Reagent Co., Ltd. (Shanghai, China). Double-distilled water (SZ-93, Shanghai Yarong Instrument Co., Ltd) was used in all the experiments.

### 2.2. Experimental procedure

The experimental procedure and apparatus have been described previously (4). In brief, ASA (0.30, 0.50, and 0.70 mmol) was dissolved in 10 mL of phosphate buffer (0.20 M), and then, the pH was adjusted to 5.0, 7.0, and 9.5 with NaOH using a pH meter (Sartorius AG, China), respectively. The mixture was then sealed in a high temperature and pressure autoclave (BE100-HP, stainless steel 316L, Shanghai LABE Instrument Co., Ltd) with quartz lining. After the reaction solution was loaded into the reactor and the reactor is assembled, nitrogen was continuously introduced from the inlet pipeline to allow air to escape from the reactor and solution, so as to ensure the whole reaction is in an oxygen-free atmosphere. The reaction temperature and time ranged from 150 to 190°C and from 30 to 150 min, respectively. The reaction temperatures were controlled using the oven, and the pressure defaulted to the saturated vapor pressure of water, ranging from 0.791 to 1.254 MPa, at the experimental temperature (29). The reaction was terminated with ice-bath water when each reaction time was reached. The experiments were carried out at least in triplicate under identical conditions to eliminate errors.

### 2.3. Measurement methods

#### 2.3.1. Determination of the ASA concentration

The concentration of ASA was determined using reverse phase high-performance liquid chromatography (HPLC) (Agilent, model 1260, Santa Clara, CA, USA) equipped with a UV diode array detector (Agilent, 1260 Infinity II DAD WR, Waldbronn, Germany) and a C_18_ column (Agilent, HC-C_18_, 3.5 μm, 4.6 mm i.d. × 100 mm, Santa Clara, CA, USA). The mobile phase was composed of water/methanol/0.1 (wt. %) and meta-phosphate (v % / v % / v %) by using the following gradient for elution. At first, the elute solution ratio was set as 8/8/84 and was changed to 50/50/0 in 20 min. Then, the ratio was changed to 8/8/84 in 6 min. During the subsequent elution, the elute solution ratio was set as 8/8/84. The whole elution time was 35 min. The flow rate was 1.0 mL/min, the column temperature was 30°C, the injection volume was 10 μL, and the detection wavelength was 243 nm (9).

The calibration curve was drawn with ASA as the external standard. The linear regression equation of ASA was as follows: *Y* = 30.10757 X+6.64471 (X is the standard solution concentration, Y is the peak area, and the correlation coefficient, *R*^2^ = 0.9998). The experiments were conducted at least three times to achieve a relative standard deviation (RSD) lower than 5 %.

#### 2.3.2. Determination of uncolored intermediate products (UIPs) and BPs

The UV absorbance and browning were measured as reported by Li et al. (9) and Zhou et al. (30). The UV absorbance of the reaction solutions was measured at 294 nm for the UIPs (31) and at 420 nm for the BPs (32) using a spectrophotometer (UV 2550, Shimadzu Co., Ltd., Kyoto, Japan). The solution was diluted using 30x−100x/0x−25x to obtain the highest optical density value, in which the absorbance was less than 1.0.

The experiments were performed at least three times to achieve a relative standard deviation (RSD) less than 5 %.

### 2.4. Kinetics

**SCHEME 1:**
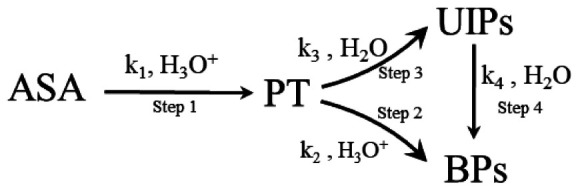
The formation of BPs from the ASA degradation.

The possible degradation mechanisms of ASA to form BPs are proposed as follows: Scheme 1.

#### 2.4.1. Kinetic model of the ASA degradation

In the reaction system, only ASA participated in the reaction as the material.

**nASA → → the final products**

As ASA was the only initial compound present at the time (t) zero in the solution loaded in the closed reactor, it was the only compound whose concentration was measured during the reaction. The following chemical equation can be written as Formula A (5).


(A) $$\text{r}=-\frac{1}{n}\,\frac{d\,\left[A\,S\,A\right]}{d\,t}=k\,{\left[A\,S\,A\right]}^{n}$$

where *r* is the velocity of reaction at a constant volume, *n* is the stoichiometric coefficient of ASA in the chemical reaction, [ASA] is the ASA concentration remaining at time *t*, *k* is the kinetic rate constant, and *n* is the pseudo-order of the reaction.

The decreasing concentration of ASA over time *t*, as presented in Formula A, could be calculated using the consumption concentration of ASA and the elapsed time *t*.

The apparent activation energy (*Ea*) was calculated using the Arrhenius equation (33), which was estimated using *ln k* and *1/T* as shown in Formula B.


(B) $$\frac{d\,\ln k}{d\,T}=\frac{E_{a}}{R\,T^{2}}$$

#### 2.4.2. Kinetic model for the formation of BPs

As one of the final products of ASA degradation, BPs are generated using two pathways. According to previous studies (6, 7, 16), first, the aldehyde pentose (PT) is generated by the ring-opening, decarboxylation reaction of ASA in step 1. Then, BPs are generated through two different pathways, Step 2 and Step 4. On the one hand, UIPs are generated through the further degradation of PT in Step 3, and then, BPs are generated subsequently in Step 4 because UIPs are the precursors of BPs (5). Step 3 and Step 4 proceed under acidic, neutral, or basic conditions. On the other hand, because PT is a polyhydroxy intermediate with an aldehyde group and its chemical structure resembles that of sugar, BPs are generated using the caramelization reaction pathway in Step 2, which occurs under acidic conditions (34).

### 2.5. Statistical analysis

The data, kinetics calculation, and correlation analysis were performed using Origin Pro 8.0 software and Microsoft office 2010. All statistical analyses were performed with at least a 95% level of confidence.

## 3. Results and discussion

### 3.1. The final color of the reaction solution

**FIGURE 1:**
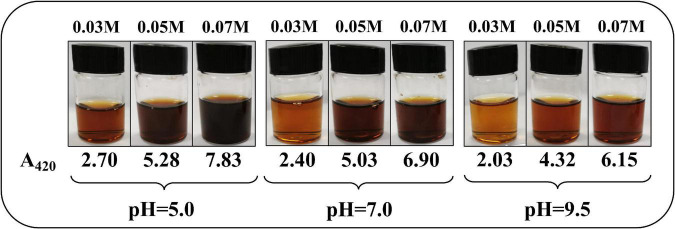
The color of the reaction solution at different concentrations of *L*-ascorbic acid (ASA) and pH values, at 190°C, 90 min.

The final color of the reaction solution is shown in Figure 1 under different concentrations of ASA and pH values when the temperature was 190°C and the time was 90 min. Intuitively, it could be seen that under different reaction conditions, the solution presented different degrees of brown.

The solution color at pH 5.0 was darker than those under other pH values, while the color of the solutions at pH 7.0 and 9.5 were very similar. The much higher concentrations of ASA resulted in much more darkening of the reaction solution, which was observed at each pH in Figure 1. However, all the reaction solutions were clear and transparent, and no precipitations were observed. In addition, it is worth noting that pH could affect the state of the chromophore, and the solution color could be changed. However, BPs were measured in a neutral environment because the solution has been greatly diluted during detection.

### 3.2. Effects of the parameters on ASA degradation

**FIGURE 2:**
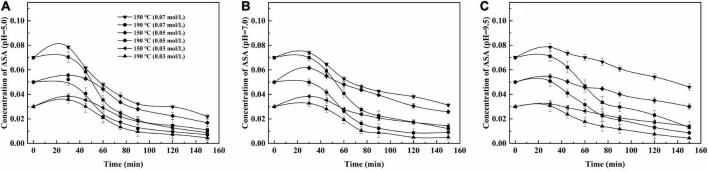
Effect of reaction parameters on the self-degradation of ASA under two temperatures. **(A)** pH = 5.0; **(B)** pH = 7.0; **(C)** pH = 9.5.

In the HCW condition, the reaction parameters, which included reaction temperature, time, the concentration of ASA, and pH, had remarkable effects on the degradation of ASA, and the results are shown in Figure 2.

The effect of temperature on the degradation of ASA was obvious. The degradation rate of ASA was faster (5, 9, 30, 35). The concentration of ASA decreased as the reaction time elapsed; the concentration of ASA reached equilibrium within the long reaction time. These data are statistically fitted to a pseudo-first-order reaction, which suggests that the value, of *n* = 1, was 1, and the values of R^2^ are shown in Table 1. The phenomena could be observed from the continuous lines shown in Figures 1, 2.

**TABLE 1:** The kinetic constant k (min^–1^) values under different reaction parameters.

pH	150°C			170°C			180°C			190°C			Ea	Ea*
	0.03M	0.05M	0.07M	0.03M	0.05M	0.07M	0.03M	0.05M	0.07M	0.03M	0.05M	0.07M	(kJ/mol)	(kJ/mol)
	(R^2^)	(R^2^)	(R^2^)	(R^2^)	(R^2^)	(R^2^)	(R^2^)	(R^2^)	(R^2^)	(R^2^)	(R^2^)	(R^2^)		
5.0	0.01279	0.01049	0.0101	0.01466	0.01218	0.01175	0.01523	0.01329	0.01303	0.01768	0.01667	0.01594	15.77	77.79
	±0.00049	±0.00014	±0.00039	±0.00093	±0.00094	±0.00049	±0.00077	±0.00087	±0.00098	±0.00020	±0.00049	±0.00078	±1.08	
	(0.9922)	(0.9777)	(0.9281)	(0.987)	(0.8688)	(0.9427)	(0.9782)	(0.9089)	(0.9611)	(0.9734)	(0.9187)	(0.925)		
7.0	0.0085	0.00736	0.0069	0.01378	0.01181	0.01089	0.01436	0.01235	0.0118	0.01739	0.01571	0.0149	31.7	46.88
	±0.00054	±0.00039	±0.00040	±0.00068	±0.00099	±0.00092	±0.00014	±0.00027	±0.00016	±0.00061	±0.00076	±0.00070	±2.41	
	(0.9693)	(0.996)	(0.9462)	(0.9679)	(0.9925)	(0.9574)	(0.9505)	(0.9886)	(0.9717)	(0.8943)	(0.853)	(0.9542)		
9.5	0.00697	0.0049	0.00439	0.01146	0.01031	0.00919	0.01361	0.0121	0.01162	0.01587	0.01478	0.0138	47.53	93.33
	±0.00060	±0.00094	±0.00077	±0.00041	±0.00017	±0.00026	±0.00023	±0.00091	±0.00041	±0.00099	±0.00015	±0.00038	±8.14	
	(0.9992)	(0.9945)	(0.9925)	(0.9894)	(0.9867)	(0.9888)	(0.9817)	(0.9824)	(0.9904)	(0.9961)	(0.9901)	(0.9773)		

*Previous work (9).

The effect of the initial concentration of ASA on the reaction was remarkable. After the reaction, the residual concentration of ASA in the solution was higher and its degradation rate was lower when the initial concentration was 0.07 M compared with those of other concentrations. When the solution pH was 5.0 and the reaction temperature was 190°C, the degradation rate of the reaction solution with the initial ASA concentration of 0.07 M was 84.4%, while the degradation rates of the reaction solutions with the initial ASA concentration of 0.05 M and 0.03 M were 86.2 and 87.3%, respectively.

The effect of pH on ASA degradation was remarkable, which can also be observed in Figure 1. The results shown in Figure 2 imply that ASA degradation consumed much faster in the acidic environment than in other conditions. When pH was 9.5, the change tendency was not obvious, and the curve of the decreased concentration of ASA became smooth. As reported previously (9), there was an enol moiety in the structure of ASA, which was prone to dehydration and isomerization reaction under acidic conditions and led to the degradation of ASA (7).

### 3.3. Relationship between the concentration of ASA and A_294 *nm*_ and A_420 *nm*_

**FIGURE 3:**
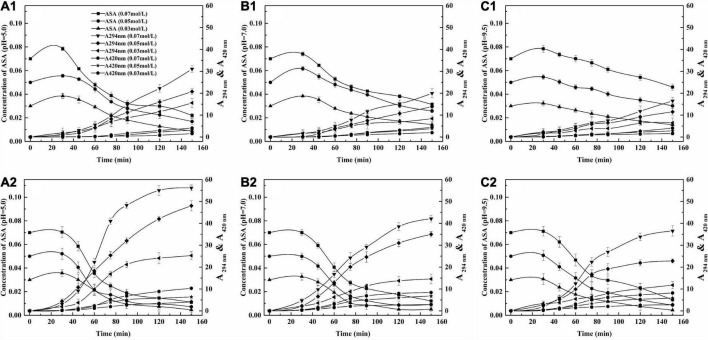
The relationship between concentration of ASA, A_294 *nm*_ and A_420 *nm*_. **(A)** pH = 5.0, **(B)** pH = 7.0, **(C)** pH = 9.5; 1, 150°C, 2, 190°C.

The relationship between the concentration of ASA and A_294 *nm*_ and A_420 *nm*_ at two temperatures 150 and 190°C and three pH values (5.0, 7.0, and 9.5) is shown in Figure 3. The results suggest that A_294 *nm*_ and A_420 *nm*_ increased as the concentration of ASA decreased at all parameters. The degree of increase in the two situations, A_294 *nm*_ and A_420 *nm*_, was different.

The concentration of ASA decreased rapidly when time ranged from 0 to 90 min, while the absorbance of the UIPs and A _294 *nm*_ increased fast, which indicates that the generation of UIPs was closely related to the consumption of ASA. The absorbance of the BPs was also increased as the A_294 *nm*_ increased. However, the increasing range of A _420 *nm*_ was not significant as that of A_294 *nm*_. That was because the UIPs are the precursors for the formation of the BPs during the ASA degradation.

When the pH was 5.0, at 150°C and the three concentrations of ASA, with increasing reaction time (30–150 min), the absorbance value of 420 nm, A_420 *nm*_, increased from 0 to 1.58, 0 to 3.15, and 0 to 4.11, respectively (Figure 3A1). However, within the same reaction time, the values of A_420 *nm*_ increased from 0 to 4.10, 0 to 6.40, and 0 to 10.33 at 190°C (Figure 3A2). The absorbances of A _294 *nm*_ and A _420 *nm*_ were less than the absorbance value at a pH of 5.0 compared with those at the pH of 7.0 and 9.5 at different concentrations of ASA. Therefore, the formation of BPs would be promoted with the higher reaction temperature and increased concentration of ASA under acidic conditions.

### 3.4. Kinetic study

#### 3.4.1. Kinetic model for ASA degradation

The degradation kinetic behavior of ASA was analyzed according to the kinetic model when the temperature ranged from 150 to 190°C and the pH value was 5.0, 7.0, and 9.5, respectively. According to the method described in a previous kinetic calculation, Formula (1), it was found that the ASA degradation complied with the pseudo-first-order, and the n in Formula 1 was 1. The degradation rate constant (*k*) under different parameters is presented in Table 1.

The kinetic data obtained from the experiment indicate that the reaction temperature and pH had a remarkable influence on *k* for ASA degradation, while the excessive concentration of ASA had no obvious effect on it. The rate constant (*k*) for ASA degradation varied from 0.00439 to 0.01279 at the lower reaction temperature, 150°C, three pH values, and the different concentrations of ASA. However, within the three pH values and the various concentrations of ASA, *k* varied from 0.01380 to 0.01768 at the higher reaction temperature, 190°C. In particular, it was found that at the same temperature and pH, *k* decreased slightly as the concentration of ASA increased, which may be due to the unique properties of water, which participated in the reaction in HCW. The water dissociation capacity is enhanced in HCW compared with conventional conditions. When the concentration of ASA was relatively low, the chance of mutual contact between ASA and water molecules under microscopic conditions was higher, and water was more likely to participate in the ASA degradation, which would accelerate the ASA degradation. In addition, the apparent activation energies (*Ea*) at pH 5.0, 7.0, and 9.5 were calculated according to the kinetic model, and the values were 15.77, 31.70, and 47.53 kJ/mol, respectively. The *Ea* for ASA degradation at pH 5.0 was much smaller than those values at pH 7.0 and 9.5, which indicates that ASA was much more easily degraded under acidic conditions to generate BPs. The *Ea* for ASA degradation in HCW was much less than those in a previous study (9). The aforementioned results suggest that water might play a non-negligible role in ASA degradation in the microenvironment of HCW, and the possible mechanism of ASA in HCW was not the same as that in conventional conditions.

#### 3.4.2. Kinetic study for the formation of BPs

According to the aforementioned proposed degradation mechanism of ASA in HCW shown in Scheme 1, the formation rate of BPs could be calculated and expressed by applying the law of mass action and is shown as follows:


(1) $$\frac{d\,\left[B\,P\,s\right]}{d\,t}=k_{2}\,\left[H_{3}\,O^{+}\right]\,\left[P\,T\right]+k_{4}\,\left[H_{2}\,O\right]\,\left[U\,I\,P\,s\right]$$

According to Scheme 1, the formation concentration of [*PT*] and [*UIPs*] could be represented by Equations 2, 3 using the steady state conditions and approximately operating (36).


(2) $$\left[P\,T\right]=\frac{k_{1}\,\left[A\,S\,A\right]\,\left[H_{3}\,O^{+}\right]}{k_{2}\,\left[H_{3}\,O^{+}\right]+k_{3}\,\left[H_{2}\,O\right]}$$

(3) $$\left[U\,I\,P\,s\right]=\frac{k_{3}\,\left[P\,T\right]}{k_{4}}$$

Substituting Equations 2, 3 to Equation 1, Equation 4 could be obtained by properly operating.


(4) $$\frac{d\,\left[B\,P\,s\right]}{d\,t}=k_{1}\,\left[H_{3}\,O^{+}\right]\,\left[A\,S\,A\right]$$

According to the law of mass, the total concentration of ASA, [*ASA*]*_*T*_*, is shown in Equation 5.


(5) $${\left[A\,S\,A\right]}_{T}=\left[A\,S\,A\right]+\left[P\,T\right]+\left[U\,I\,P\,s\right]+\left[B\,P\,s\right]$$

BPs are the final substances from the degradation of ASA, and they are macromolecular polymers and their solubility in water is limited. However, no materials could be observed, and the reaction solution was clear (Figure 1). The phenomena suggest that the number of BPs generated from the degradation of ASA was very limited. Compared with the concentration of ASA and PT, as the UIPs were the precursors of BPs from the aforementioned experimental results, the concentration of the BPs could be negligible. Equation 5 could be simplified as follows:


(6) $${\left[A\,S\,A\right]}_{T}=\left[A\,S\,A\right]+\left[P\,T\right]+\left[U\,I\,P\,s\right]$$

Substitute Equations 2, 3 into Equation 6 to obtain the following equation,


(7) $${\left[A\,S\,A\right]}_{T}=\frac{k_{1}\,k_{4}\,\left[H_{3}\,O^{+}\right]+k_{1}\,k_{3}\,\left[H_{3}\,O^{+}\right]+k_{2}\,k_{4}\,\left[H_{3}\,O^{+}\right]+k_{3}\,k_{4}\,\left[H_{2}\,O\right]\,\left[A\,S\,A\right]}{k_{4}\,\left(k_{2}\,\left[H_{3}\,O^{+}\right]+k_{3}\,\left[H_{2}\,O\right]\right)}$$

Swap [*ASA*] and [*ASA*]*_*T*_* in Equation 7 to obtain Equation 8, which is shown as follows:


(8) $$\left[A\,S\,A\right]=\frac{k_{4}\,\left(k_{2}\,\left[H_{3}\,O^{+}\right]+k_{3}\,\left[H_{2}\,O\right]\right)\,{\left[A\,S\,A\right]}_{T}}{k_{1}\,k_{4}\,\left[H_{3}\,O^{+}\right]+k_{1}\,k_{3}\,\left[H_{3}\,O^{+}\right]+k_{2}\,k_{4}\,\left[H_{3}\,O^{+}\right]+k_{3}\,k_{4}\,\left[H_{2}\,O\right]}$$

Substitute Equation 8 into Equation 4 to obtain Equation 9 as follows:


(9) $$\frac{d\,\left[B\,P\,s\right]}{d\,t}=\frac{k_{1}\,k_{4}\,\left[H_{3}\,O^{+}\right]\,\left(k_{2}\,\left[H_{3}\,O^{+}\right]+k_{3}\,\left[H_{2}\,O\right]\right)\,{\left[A\,S\,A\right]}_{T}}{k_{1}\,k_{4}\,\left[H_{3}\,O^{+}\right]+k_{1}\,k_{3}\,\left[H_{3}\,O^{+}\right]+k_{2}\,k_{4}\,\left[H_{3}\,O^{+}\right]+k_{3}\,k_{4}\,\left[H_{2}\,O\right]}$$

In the HCW reaction system, [*H*_3_*O*^+^] was a very small value (10^–9.5^–10^–5.0^) because the reaction solution pH was set at 5.0, 7.0, and 9.5. However, there was a large amount of water in the reaction system as *k_1_, k_2_, k_3_*, and *k*_4_ could not arrive at several orders of magnitude. Therefore, *k*_1_*k*_4_[*H*_3_*O*^+^], *k*_1_*k*_3_[*H*_3_*O*^+^], and *k*_2_*k*_4_[*H*_3_*O*^+^] could be neglected compared with *k*_3_*k*_4_[*H*_2_*O*], so Equation 9 can be simplified as Equation 10.


(10) $$\frac{d\,\left[B\,P\,s\right]}{d\,t}=\left(\frac{k_{2}\,\left[H_{3}\,O^{+}\right]}{k_{3}\,\left[H_{2}\,O\right]}+1\right)\,k_{1}\,\left[H_{3}\,O^{+}\right]\,{\left[A\,S\,A\right]}_{T}$$

Though the water was consumed in the HCW system and it participated in the reaction as the raw substance, its amount was huge compared with the concentration of ASA. The concentration of water was much more than the other substances and could be regarded as a constant value. Therefore, the product *k_3_’*, which is [*H*_2_*O*] times *k*_3_, is also a constant. Thus, Equation 10 can be simplified as Equation 11,


(11) $$\frac{d\,\left[B\,P\,s\right]}{d\,t}=\left(\frac{k_{2}\,\left[H_{3}\,O^{+}\right]}{k_{3}{}^{\prime }}+1\right)\,k_{1}\,\left[H_{3}\,O^{+}\right]\,{\left[A\,S\,A\right]}_{T}$$

[*ASA*]*_*T*_* is also a constant in a settled reaction system according to the material balance principle, which meant that the browning rate was independent of the concentration of ASA. In addition, the browning rate was also related to the concentration of *H_3_O^+^* according to Equation 11, which indicates that the formation rate of BPs was greatly affected by the pH value of the reaction system. According to the results shown in Figure 1, the solution color change from the degradation of ASA was closely related to the primary solution pH, which conformed to the result of Equation 11. Once the concentration of hydrogen ions was determined, the formation rate of BPs could be determined in the reaction.

Combining Scheme 1 and Equation 11, it was found that the two different pathways, Step 2 and Step 3, competed with each other to generate BPs. The values of *k*_2_ and *k_3_’* in the system when the concentration of *H_3_O^+^* is a certain value determine which pathway is generated for the formation of the BPs.

## 4. The mechanism of ASA degradation and the formation of BPs

**FIGURE 4:**
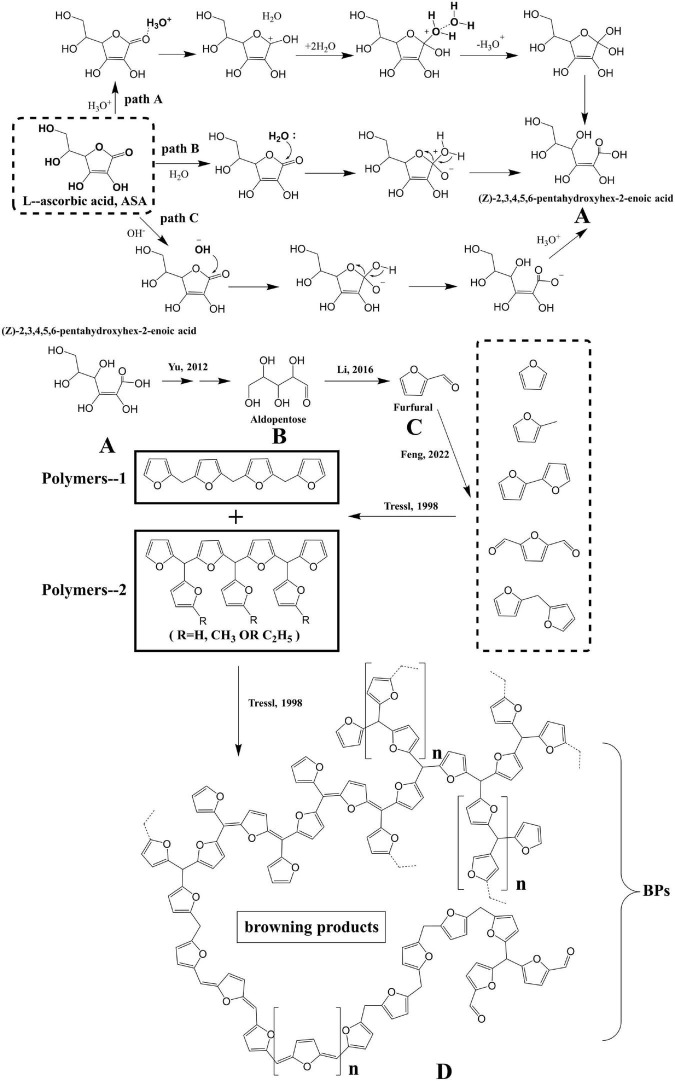
The possible mechanism of hydrolysis mechanism of ASA and formation of BPs. **(A)** (Z)-2,3,4,5,6-pentahydroxyhex-2-enoic acid, **(B)** aldopentose, **(C)** furfural, and **(D)** browning products.

According to the experimental results and the substances detected from the reaction (4), paths A, B, and C, and the possible mechanisms for the degradation of ASA in HCW were proposed and are shown in Figure 4.

The presence of the lactone structure and poly hydroxyl groups in ASA means that the structure of ASA could be transformed through ring-opening or isomerization reactions. Under the HCW condition, the dissociation constant of water increased with increasing temperature, which caused more hydrogen and hydroxide ions to be generated. Based on the reasons mentioned earlier, the degradation mechanism of ASA may be different in HCW compared with that in the ambient condition (37).

From the experimental results, the collected substances, and the organic chemistry theory, there are three possible mechanisms for the structural transformation of ASA under different acidic or basic conditions.

For pathway A, when the reaction solution is in the acidic condition, the carbonyl oxygen atom in ASA is protonated by hydronium ions, coming from HCW, and then an unstable intermediate structure is generated. The intermediate structure could become stable after being surrounded by water molecular clusters in HCW, and finally, it undergoes intramolecular proton transfer to generate product A (Figure 4). For pathway B, the lactone carbonyl of ASA is also directly attacked by the nucleophilic of water to undergo the nucleophilic addition reaction due to the lower dielectric constant and weaker hydrogen bonding of water molecules in HCW (Figure 4). For pathway C, because there are also abundant hydroxide ions in HCW, the structure of lactone in ASA can also be attacked by hydroxide ion, resulting in a nucleophilic addition-elimination reaction (Figure 4). The three hydrolysis mechanisms of ASA may exist simultaneously in HCW, and they are the competing reactions. However, regardless of which of the pathway mechanisms is used for the hydrolysis of ASA, the product A, (Z)-2,3,4,5-pentahydroxyhex-2-enoic acid, is ultimately generated.

Product A undergoes further decarboxylation and dehydration reaction to generate product B, aldopentose. Then, a large amount of furfural, product C, including the derivatives of furan, is generated from the decomposition of B, which has been reported in a previous study (4). Owing to the abundance of furan derivatives, they react with each other, and then the macromolecule, product D, is generated eventually.

At present, there are some viewpoints about the structure of BPs generated from the Maillard reaction (38–40). One of the views is that BPs are mainly composed of repeating units of furan, and they finally form the Maillard reaction product through the polycondensation reaction (38). According to the aforementioned experimental results, it could be concluded that the BPs generated from the degradation of ASA may conform to the view mentioned previously.

BPs are formed by the polymerization of furan or furan derivatives, the main low molecular weight products generated from the degradation of ASA. Furan or furan derivatives are polymerized into polymer-1 and polymer-2, respectively. The structures of polymer-1 and polymer-2 merely represented different structures or substructures of brown polymer. In fact, during the complex degradation process of ASA, many different polycondensation reactions occurred simultaneously and produced the complex BPs, product D, which was also generally defined as BPs.

## 5. Conclusion

From the experimental results, the degradation behavior of ASA and the formation mechanism of BP in HCW were discussed and presented. Compared with neutral or alkaline environments, ASA was more easily degraded under acidic conditions, which also accelerated the formation of BPs. In addition, the browning reaction was significantly accelerated by increasing the reaction temperature or prolonging the reaction time. The degradation of ASA followed the pseudo-first-order kinetics, and the degradation of the *Ea* of ASA was the least in the acidic system. The formation of BPs closely depended on the concentration of hydrogen ions. Finally, the possible degradation mechanism of ASA and the formation mechanism of BPs were proposed based on the experimental results. It was found that there were several hydrolysis mechanisms of ASA degradation. BPs might be the complex macromolecule formed by the condensation of various furan derivatives.

## Data availability statement

The original contributions presented in this study are included in the article/supplementary material, further inquiries can be directed to the corresponding author.

## Author contributions

LF: data collection and curation, formal analysis, and writing the original draft. YY: supervision, funding acquisition, project administration, reviewing, and editing. Y-TX, S-SL, and XP: data collection and curation. SH and A-NY: supervision and funding acquisition. All authors contributed to the article and approved the submitted version.

## References

[1] Bosch V Cilla A García-Llatas G Gilabert V Boix R Alegría A. Kinetics of ascorbic acid degradation in fruit-based infant foods during storage. *J Food Eng.* (2013) 116:298–303. 10.1016/j.jfoodeng.2012.12.003

[2] Kim AN Lee KY Rahman MS Kim HJ Kerr WL Choi SG. Thermal treatment of apple puree under oxygen-free condition: effect on phenolic compounds, ascorbic acid, antioxidant activities, color, and enzyme activities. *Food Biosci.* (2020) 39:100802. 10.1016/j.fbio.2020.100802

[3] Ozcelik M Ambros S Morais SIF Kulozik U. Storage stability of dried raspberry foam as a snack product: effect of foam structure and microwave-assisted freeze drying on the stability of plant bioactives and ascorbic acid. *J Food Eng.* (2020) 270:109779. 10.1016/j.jfoodeng.2019.109779

[4] Feng L Yang Y Liu S-S Tan D-Y Tan C Yu A-N. The study of volatile products formation from the self-degradation of L-ascorbic acid in hot compressed water. *Food Chem.* (2022) 2022:131155. 10.1016/j.foodchem.2021.131155 34571410

[5] Yang Y Li Y Feng L Yu A-N Sun B-G Liu Y-P. The effects of reaction parameters on the non-enzymatic browning reaction between *L*-ascorbic acid and glycine. *Int J Food Eng.* (2021) 17:49–56. 10.1515/ijfe-2019-0189

[6] Kurata T Sakurai Y. Degradation of L-ascorbic acid and mechanism of nonenzymic browning reaction. *Part II Agric Biol Chem.* (1967) 31:177–84. 10.1271/bbb1961.31.177

[7] Kurata T Wakabayashi H Sakurai Y. Degradation of L-ascorbic acid and mechanism of nonenzymic browning reaction. *Part I Agric Biol Chem.* (1967) 31:101–5. 10.1080/00021369.1967.10858773

[8] Lee HS Nagy S. Quality changes and nonenzymic browning intermediates in grapefruit juice during storage. *J Food Sci.* (1988) 53:168–72.

[9] Li Y Yang Y Yu A-N Wang K. Effects of reaction parameters on self-degradation of *L*-ascorbic acid and self-degradation kinetics. *Food Sci Biotechnol.* (2016) 25:97–104. 10.1007/s10068-016-0014-x 30263242 PMC6049385

[10] Marfil PHM Santos EM Telis VRN. Ascorbic acid degradation kinetics in tomatoes at different drying conditions. *LWT-Food Sci Technol.* (2008) 41:1642–7. 10.1016/j.lwt.2007.11.003

[11] Nagy S Smoot JM. Temperature and storage effects on percent retention and percent U.S. recommended dietary of vitamin C in canned single-strength orange juice. *J Agric Food Chem.* (1977) 25:135–8. 10.1021/jf60209a031 1002912

[12] Smoot JM Nagy S. Effects of storage temperature and duration on total vitamin C content of canned single-strength grapefruit juice. *J Agric Food Chem.* (1980) 28:417–21. 10.1021/jf60228a050 7391377

[13] Tiwari BK Donnell CPO Muthukumarappan K Cullen PJ. Ascorbic acid degradation kinetics of sonicated orange juice during storage and comparison with thermally pasteurised juice. *LWT-Food Sci Technol.* (2009) 42:700–4. 10.1016/j.lwt.2008.10.009

[14] Villota R Karel M. Prediction of ascorbic acid retention during drying. I. Moisture and temperature distribution in a model system. *J Food Process Preserv.* (1980) 4:111–34.

[15] Wong M Stanton DW. Nonenzymic browning in kiwifruit juice concentrate systems during storage. *J Food Sci.* (1989) 54:669–73.

[16] Yu A-N Tan Z-W Wang F-S. Mechanism of formation of sulphur aroma compounds from *L*-ascorbic acid and *L*-cysteine during the maillard reaction. *Food Chem.* (2012) 132:1316–23. 10.1016/j.foodchem.2011.11.111 29243617

[17] Yu A-N Tan Z-W Wang F-S. Mechanistic studies on the formation of pyrazines by maillard reaction between *L*-ascorbic acid and *L*-glutamic acid. *LWT Food Sci Technol.* (2013) 50:64–71. 10.1016/j.lwt.2012.07.001

[18] Yu A-N Zhang A-D. The effect of pH on the formation of aroma compounds produced by heating a model system containing *L*-ascorbic acid with *L*-threonine/*L*-serine. *Food Chem.* (2010) 119:214–9. 10.1016/j.foodchem.2009.06.026

[19] Liu H-M Wang F-Y Liu Y-L. Hot-compressed water extraction of polysaccharides from soy hulls. *Food Chem.* (2016) 202:104–9. 10.1016/j.foodchem.2016.01.129 26920272

[20] Mokgadi J Torto N Turner C. Pressurized hot water extraction of alkaloids in goldenseal. *Am J Anal Chem.* (2013) 4:398–403. 10.4236/ajac.2013.48050

[21] Cho YJ Getachew AT Park JS Lim CT Lee HJ Chun BS. Influence of temperature on decomposition reaction of compressed hot water to valorize achatina fulica as a functional material. *Food Bioprod Process.* (2020) 122:89–97. 10.1016/j.fbp.2020.03.008

[22] David N Phillip P Byung SC. Optimization and kinetics modeling of okara isoflavones extraction using subcritical water. *Food Chem.* (2019) 295:613–21. 10.1016/j.foodchem.2019.05.129 31174803

[23] Gbashi S Adebo O Piater L Madala NE Phoku JZ Njobeh PB. Subcritical water extraction of biological materials. *Separation Purif Rev.* (2016) 46:21–34. 10.1080/15422119.2016.1170035

[24] Yang Y Duan P-G Wang Y-Y Dai L-Y. Additives assisted catalytic cyclo-dehydration of diethylene glycol in near-critical water. *Chem Eng Process Process Intens.* (2008) 47:2402–7. 10.1016/j.cep.2007.12.011

[25] Akiya N Savage PE. Roles of water for chemical reactions in high-temperature water. *Chem Rev.* (2002) 102:2725–50. 10.1021/cr000668w 12175266

[26] Shitu A Izhar S Tahir TM. Sub-critical water as a green solvent for production of valuable materials from agricultural waste biomass: a review of recent work. *Global J Environ Sci Manage.* (2015) 1:255–64. 10.7508/gjesm.2015.03.008

[27] Torres MD Fradinho P Rodríguez P Falqué E Santos V Domínguez H. Biorefinery concept for discarded potatoes: recovery of starch and bioactive compounds. *J Food Eng.* (2020) 275:109886. 10.1016/j.jfoodeng.2019.109886

[28] Takada M Minami E Saka S. Decomposition behaviors of the lignocellulosics as treated by semi-flow hot-compressed water. *J Supercr Fluids.* (2017) 133:566–72. 10.1016/j.supflu.2017.07.007

[29] Dinjus E Kruse A. Hot compressed water-a suitable and sustainable solvent and reaction medium? *J Phys Condensed Matter.* (2004) 16:1161–9. 10.1088/0953-8984/16/14/026

[30] Zhou Y-Y Li Y Yu A-N. The effects of reactants ratios, reaction temperatures and times on Maillard reaction products of the *L*-ascorbic acid/*L*-glutamic acid system. *Food Sci Technol.* (2016) 36:268–74. 10.1590/1678-457X.02415

[31] Ajandouz EH Tchiakpe LS Ore FD Benajiba A Puigserver A. Effects of pH on caramelization and Maillard reaction kinetics in fructose-lysine model systems. *J Food Sci.* (2001) 66:926–31. 10.1111/j.1365-2621.2001.tb08213.x

[32] Lertittikul W Benjakul S Tanaka M. Characteristics and antioxidative activity of Maillard reaction products from a porcine plasma protein-glucose model system as influenced by pH. *Food Chem.* (2007) 100:669–77. 10.1016/j.foodchem.2005.09.085

[33] Yu A-N Li Y Yang Y Yu K. The browning kinetics of the non-enzymatic browning reaction in *L*-ascorbic acid/basic amino acid systems. *Food Sci Technol (Campinas).* (2017) 38:537–42. 10.1590/1678-457x.08717

[34] Buera M Chirife J Resnik SL Lozano RD. Nonenzymatic browning in liquid model systems of high water activity: kinetics of color changes due to caramelization of various single sugars. *J Food Sci.* (2010) 52:1059–62. 10.1111/j.1365-2621.1987.tb14276.x

[35] Yu A-N Zhou Y-Y Yang Y-N. Kinetics of browning and correlations between browning degree and pyrazine compounds in *L*-ascorbic acid/acidic amino acid model systems. *Food Chem.* (2017) 221:1678–84. 10.1016/j.foodchem.2016.10.119 27979146

[36] Adamson AW. *A Textbook of Physical Chemistry. Kinetics of Reactions in Solution.* 2th ed. New York, NY: Jovanovich Publishing (1973). p. 604–52.

[37] Oka H Yamago S Yoshida J Kajimoto O. Evidence for a hydroxide ion catalyzed pathway in ester hydrolysis in supercritical water. *Angewandte Chemie-Int Ed.* (2002) 41:623–5. 10.1002/1521-3757(20020215)114:43.0.CO;2-6

[38] Tressl R Wondrak GT Garbe L-A Kruger R-P Rewicki D. Pentoses and hexoses as sources of new melanoidin-like Maillard polymers. *J Agric Food Chem.* (1998) 46:1765–76. 10.1021/jf970973r 10554203

[39] Hofmann T. Studies on melanoidin-type colorants generated from the Maillard reaction of protein-bound lysine and furan-2-carboxaldehyde – chemical characterisation of a red coloured domaine. *Eur Food Res Technol.* (1998) 206:251–8. 10.1007/s002170050253

[40] Cammerer B Jalyschko V Kroh LW. Carbohydrate structures as part of the melanoidin skeleton. *J Agric Food Chem.* (2002) 1245:269–73. 10.1016/S0531-5131(02)00890-7 11902960

